# ASPP: a new family of oncogenes and tumour suppressor genes

**DOI:** 10.1038/sj.bjc.6603525

**Published:** 2007-01-09

**Authors:** A Sullivan, X Lu

**Affiliations:** 1 Ludwig Institute for Cancer Research, University College London, 91 Riding House Street, London W1W 7BS, UK

**Keywords:** ASPP, p53, apoptosis

## Abstract

The apoptosis stimulating proteins of p53 (ASPP) family consists of three members, ASPP1, ASPP2 and iASPP. They bind to proteins that are key players in controlling apoptosis (p53, Bcl-2 and RelA/p65) and cell growth (APCL, PP1). So far, the best-known function of the ASPP family members is their ability to regulate the apoptotic function of p53 and its family members, p63 and p73. Biochemical and genetic evidence has shown that ASPP1 and ASPP2 activate, whereas iASPP inhibits, the apoptotic but not the cell-cycle arrest function of p53. The p53 tumour suppressor gene, one of the most frequently mutated genes in human cancer, is capable of suppressing tumour growth through its ability to induce apoptosis or cell-cycle arrest. Thus, the ASPP family of proteins helps to determine how cells choose to die and may therefore be a novel target for cancer therapy.

The recently discovered Apoptosis Stimulating Proteins of p53 (ASPP) family consists of three members, ASPP1, ASPP2 and iASPP. The most evolutionary conserved member, iASPP, is the inhibitory form and the only ASPP family member identified in *Caenorhabditis elegans* (Bergamaschi *et al*, 2003b). Mammals, including mice and humans, have evolved two additional members, ASPP1 and ASPP2 (Samuels-Lev *et al*, 2001). All three proteins share sequence similarity in their C-termini which contain their signature sequences of Ankyrin repeats, SH3 domain and Proline-rich region (Figure 1). Thus, ASPP also stands for Ankyrin repeats, SH3 domain, and Proline-rich region containing Protein. The C-terminus is the preferred binding site for ASPP partners including p53 (Gorina and Pavletich, 1996), RelA/p65 (subunit 3 of nuclear factor-*κ*B) (Yang *et al*, 1999a), Bcl-2 (Naumovski and Cleary, 1996), adenomatous polyposis coli-like (APCL) (Nakagawa *et al*, 2000), Hepatitis-C core protein (Cao *et al*, 2004), amyloid-*β*-precursor protein-binding protein 1 (APPBP1) (Chen *et al*, 2003), YES-associated protein-1 (YAP1) (Espanel and Sudol, 2001), Sp1 (Ohno *et al*, 1997) and SAM68 (Thornton *et al*, 2006). The N-terminus is only conserved in the pro-apoptotic members, ASPP1 and ASPP2.

To date, the most well-known function of the ASPP family of proteins is their ability to regulate apoptosis mediated by p53 and its family members, p63 and p73 (Bergamaschi *et al*, 2004). ASPP1 and ASPP2 enhance the ability of p53 to stimulate specifically the expression of pro-apoptotic target genes but not genes involved in cell-cycle arrest (Samuels-Lev *et al*, 2001; Bergamaschi *et al*, 2004), whereas iASPP inhibits p53-mediated apoptosis (Bergamaschi *et al*, 2003b). This implies that iASPP can compete with ASPP1 and ASPP2 for binding to p53, thereby inhibiting the ability of ASPP1 and ASPP2 to stimulate the apoptotic function of p53.

The p53 tumour suppressor plays an important role in regulating the proliferation of cells through either cell-cycle arrest or apoptosis (Rowan *et al*, 1996). p53 has been shown to be lost or mutated in over half of human cancers (http://www-p53.iarc.fr/). In the other 50% of tumours, where wild-type p53 is intact, the regulation of the p53 pathway is abnormal. Understanding why p53 is unable to perform its role as a tumour suppressor in these wild-type tumours has been the focus of extensive research. It is hoped that restoration of the tumour suppressor and apoptotic functions of p53 in tumours expressing wild-type p53 will enable the development of new therapeutic strategies in the treatment of cancer. In this review, we will focus on the recent understanding of the biological function of the ASPP family of proteins and their potential role in tumorigenesis.

## iASPP IS A PROTO-ONCOGENE WHEREAS ASPP1 AND ASPP2 ARE TUMOUR SUPPRESSORS

Depletion of the only homologue of the ASPP family, iASPP, in *C. elegans* by RNAi specifically increased the number of apoptotic germ cell corpses (Bergamaschi *et al*, 2003b). Because of this resulting phenotype, the gene was termed *ape-1* for *ap*optosis *e*nhancer-*1*, whose gene product is Ce-iASPP. These studies provided the initial genetic evidence implicating iASPP as an antiapoptotic gene. This unique phenotype further revealed that Ce-iASPP is a key inhibitor of p53 in *C. elegans* as RNAi of iASPP failed to show any effect in p53 null worms. Furthermore, Ce-iASPP was identified as an inhibitor of p53 in a library screen for p53 inhibitors (Lettre *et al*, 2004). Therefore, Ce-iASPP is one of the most conserved inhibitors of p53 identified so far.

Unlike iASPP, ASPP2 is pro-apoptotic and is the most well-characterised member of the ASPP family. Existing studies have suggested that ASPP2 plays a central role in the regulation of apoptosis and cell growth. The most compelling evidence comes from the ASPP2 knockout mouse study (Vives *et al*, 2006). The group examined the role of ASPP2 in tumorigenesis and its relationship with p53 tumour suppression in homozygous or heterozygous mice lacking ASPP2 or ASPP2 and p53 alleles. ASPP2 knockout mice exhibit postnatal lethality. ASPP2 heterozygous mice have 45% tumour incidence over their lifespans, whereas wild-type mice have 15% tumour incidence. Compared to the wild-type mice, the heterozygous mice develop twice as many spontaneous lymphomas, as well as rhabdomyosarcomas and squamous cell carcinomas. The ASPP2 heterozygous mice also have an increased susceptibility to the onset of tumour development in response to ionising radiation. The failure to generate a p53 and ASPP2 double knockout mouse demonstrates that ASPP2 and p53 deficiency are synthetic lethal. This phenotype also suggests that, genetically, ASPP2 shares overlapping functions with p53 in mouse development. Consistent with this, a combination of p53 and ASPP2 heterozygosity accelerated the onset of tumour development. The mouse model demonstrates unequivocally that ASPP2 is a tumour suppressor that functions as an activator of the tumour suppressor function of p53.

Biochemically, ASPP2-mediated apoptosis has been shown to be inhibited by nuclear factor-*κ*B and Bcl-2 family proteins, suggesting that the antiapoptotic functions of these proteins result from blocking the pro-apoptotic function of ASPP2 (Yang *et al*, 1999b; Takahashi *et al*, 2005). The observation that ASPP2 can localise to the mitochondria suggests that ASPP2 might play a role in regulating apoptosis through a mitochondrial-dependent pathway (Takahashi *et al*, 2005). Furthermore, treatment of wild-type and p53 null cell lines with ASPP1 and ASPP2 antisense RNA reduces apoptosis induced by DNA-damaging agents such as cisplatin, etoposide and UV (Samuels-Lev *et al*, 2001; Bergamaschi *et al*, 2003b). Cell lines stably expressing iASPP have been produced and are more resistant to apoptosis induced by cisplastin or UV compared to the parental cell lines. In addition, co-expression of ASPP1 and ASPP2 inhibits, whereas expression of iASPP enhances, the transforming activity of the oncogenes RAS and E1A in rat embryo fibroblasts, further implicating ASPP1 and ASPP2 as tumour suppressors, whereas iASPP is likely to be a proto-oncogene (Bergamaschi *et al*, 2003b).

## ALTERED ASPP EXPRESSION IN HUMAN CANCERS IS LINKED TO TUMOUR PROGRESSION

Initial evidence suggesting the involvement of ASPP in human cancer came from the analysis of the crystal structure of the C-terminal ankyrin repeats and SH3 domains of ASPP2 and the DNA-binding domain of p53. The amino acids in p53 responsible for binding to ASPP2, namely 178His, 181Arg, 243Met and 247Arg, are all mutated to a relatively high frequency in human cancer (Gorina and Pavletich, 1996). Importantly, the two most frequently mutated residues, 248Arg and 273Arg, contact both DNA and ASPP2.

Recent studies have shown altered expression of the ASPP family members in a variety of human cancers. The expression of ASPP1 and ASPP2 is frequently downregulated in human breast tumours (Samuels-Lev *et al*, 2001) and tumour cell lines (Liu *et al*, 2005) expressing wild-type p53, suggesting that there is a selective advantage for loss of ASPP1 and ASPP2 expression in human breast tumours expressing wild-type p53. Reduced levels of ASPP2 have also been detected in human lung cancer cell lines (Mori *et al*, 2000), and ASPP1 downregulation was observed in one non-small-cell lung carcinoma cell line and two mesothelioma cell lines (Mori *et al*, 2004). Furthermore, in leukaemia cancer cell lines a concomitant downregulation of ASPP1 with an upregulation of iASPP has been observed, suggesting that abnormal ASPP expression could be an important step in the formation of human haematopoietic neoplasms and might be a useful molecular marker for the diagnosis of leukaemia (Liu *et al*, 2004). Significantly higher levels of iASPP were detected in human breast carcinomas (Bergamaschi *et al*, 2003a), leukaemia cell lines and acute leukaemias (AL) (Zhang *et al*, 2005). Interestingly, the level of iASPP gene expression was significantly higher in AL than in cells from normal donors or AL patients in complete remission, suggesting that overexpression of iASPP may promote leukaemogenesis and/or disease progression of AL (Zhang *et al*, 2005).

Other studies have linked reduced ASPP2 levels with poor prognosis and metastasis. A lowered ASPP2 expression was linked to a poor clinical outcome in diffuse large B-cell lymphoma (Lossos *et al*, 2002) and with poor distant recurrence-free survival in breast cancer patients (Cobleigh *et al*, 2005). Reduced expression of ASPP2 has also been observed in a microarray study of metastatic breast cancer samples compared to non-metastatic samples (Sgroi *et al*, 1999), suggesting that ASPP2 might be associated with breast cancer progression. Furthermore, an association between the ASPP2 (*TP53BP2*) locus and gastric cancer susceptibility has also been reported (Ju *et al*, 2005). Four single nucleotide polymorphisms (g.206692C>T, g.198267A>T, g.164895G>A and g.152389A>T) within *TP53BP2* showed a significant link with gastric cancer susceptibility.

## REGULATION OF ASPP EXPRESSION

**Figure 2:**
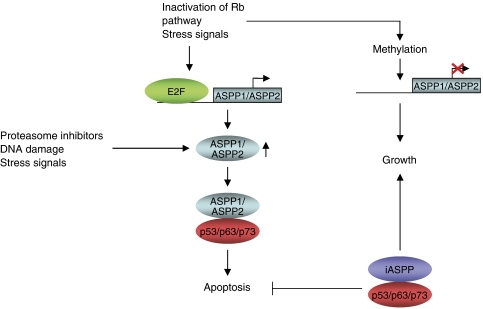
Regulation of ASPP family of proteins. Stress signals including DNA damage, upregulation of the E2F transcription factors and inhibition of the proteasome activate ASPP1 and ASPP2 to interact with p53 family members and induce apoptosis. iASPP prevents this, instead allowing cell proliferation to occur. Gene silencing of ASPP1 and ASPP2 by methylation also prevents p53-dependent apoptosis.

Our current understanding is that three main mechanisms are involved in the regulation of the ASPP family members: inactivation and gene silencing by methylation, activation by E2F transcription factors and regulation of ASPP2 at the protein level. The mechanism of action and the regulation of the ASPP family members in tumour cells is summarised in Figure 2.

### ASPP1 and ASPP2 gene silencing

DNA methylation is the main epigenetic modification in humans, and changes in the methylation status of genes play an important role in tumorigenesis. Aberrant methylation of normally unmethylated CpG islands in the promoters of various tumour suppressor genes has been reported (Merlo *et al*, 1995). Initial evidence for methylation-dependent regulation of ASPP2 expression came from the observation that methyl-CpG-binding protein 1 (MBD1) is required for the formation of the CAF-1/MBD1/SETDB1 complex, which facilitates methylation during replication-coupled chromatin assembly (Sarraf and Stancheva, 2004). In the absence of MBD1, methylation at multiple genomic loci is lost. This results in the activation of the ASPP2 gene, which is normally repressed by MBD1, demonstrating that ASPP2 expression is regulated by methylation.

Furthermore, hypermethylation of the CpG islands in the 5′ untranslated regions of the ASPP1 and ASPP2 genes and reduced expression of ASPP1 and ASPP2 is reported in HEPG-2, MCF-7 and A549 cell lines which express wild-type p53 (Liu *et al*, 2005). In contrast, in normal fibroblasts, the ASPP1 and ASPP2 genes are unmethylated. This suggests that epigenetic changes of ASPP1 and ASPP2 occur in tumours that are wild type for p53 and that downregulation of ASPP1 and ASPP2 expression correlates with abnormal methylation of the ASPP1 and ASPP2 genes. Expression of ASPP1 in acute lymphoblastic leukaemia (ALL) is also significantly reduced as a consequence of the hypermethylation of the ASPP1 gene promoter (Agirre *et al*, 2006). In addition, methylation of ASPP1 is significantly higher in adult compared to childhood ALL, and T-cell compared to B-cell ALL. Relapse rate and mortality are significantly higher in patients expressing methylated ASPP1. Finally, methylation of the ASPP1 gene promoter is indicative of a poor prognosis in ALL patients. Consistent with these findings, another study shows aberrant methylation of ASPP1 in 34% of the 50 patients with *de novo* T-cell ALL (Roman-Gomez *et al*, 2005).

### ASPP1 and ASPP2 are transcriptional targets of E2F1

The E2F1 transcription factor is a downstream target of the tumour suppressor retinoblastoma (Rb). When activated, E2F1 induces cell proliferation. E2F1 can also mediate apoptosis via p53-dependent and independent mechanisms. ASPP1 and ASPP2 have been identified as transcriptional targets of E2F, and increased expression of E2F-1 was shown to induce the expression of ASPP1 and ASPP2 at both mRNA and protein levels (Stanelle *et al*, 2002; Chen *et al*, 2005; Fogal *et al*, 2005; Hershko *et al*, 2005). Many tumour types exhibit defective Rb pathways and consequently an increased E2F activity. As a result, they also have an increased expression of ASPP1, ASPP2 or both, thereby enhancing the cellular sensitivity to p53-mediated apoptosis. This may be one possible explanation as to why tumour cells are more sensitive to p53-dependent apoptosis than primary cells.

### Regulation of ASPP2 expression at the protein level

The proteasome, a major component of the ubiquitin-mediated protein degradation pathway, has recently become a therapeutic target in cancer therapy. Treatment of cells with bortezomib, a specific inhibitor of the proteasome which is clinically active in different tumour types, increased ASPP2 protein and protein half-life but not RNA levels (Zhu *et al*, 2005). Treatment with anthracycline, a chemotherapy agent that can induce DNA damage and inhibit the proteasome, also increased ASPP2 protein but not RNA levels. In addition, knockdown of ASPP2 by siRNA attenuated bortezomib-induced apoptosis in wild-type p53 cells. Importantly, the central region of ASPP2 is ubiquitinated. Taken together, these data demonstrate that ASPP2 is a direct proteasomal substrate, and that proteasomal degradation modulates ASPP2 protein levels and apoptotic function. Furthermore, post-translational control of ASPP2 following DNA damage and stress induction may be an important mechanism by which ASPP2 stimulates apoptosis under these conditions.

## IASPP MODULATES THE APOPTOTIC FUNCTION OF P53 CODON 72 POLYMORPHIC VARIANTS

The most common p53 polymorphism whereby either an arginine (p53Arg72) or a proline (p53Pro72) is expressed at codon 72 only exists in humans and p53Arg72 is human-specific. The frequency of the allele encoding p53Pro72 is closely linked to latitude and is much higher in populations living near the equator suggesting that p53Pro72 is selected in an environment with high levels of UV light (Sjalander *et al*, 1995). A novel mechanism by which the ASPP family members selectively regulate the apoptotic function of p53Pro72 and p53Arg72 has been described (Bergamaschi *et al*, 2006). iASPP has a higher binding affinity to p53Pro72 than to p53Arg72, and preferentially modulates the apoptotic potential of p53Pro72 over p53Arg72. Furthermore, p53Arg72 is more active than p53Pro72 in the induction of apoptosis, and the greater apoptosis inducing activity of p53Arg72 results from its ability to escape iASPP inhibition. Thus, the most efficient way to inactivate the apoptotic function of p53Arg72 in human tumorigenesis is by intragenic mutation. An alternative way to inactivate the p53Pro72 isoform is by reduced expression of ASPP1, ASPP2 or overexpression of iASPP in addition to mutation in p53 itself. Therefore, the ASPP family provides an additional level of regulation of p53. This work suggests that the expression levels of the ASPP family members taken together with the expression of polymorphic p53 variants can provide clues about cancer susceptibility, prognosis and therapy.

## CONCLUSION

Inactivation of ASPP1 and ASPP2, proteins that cooperate with p53 to induce apoptosis, and activation of the inhibitor of p53, iASPP, appear to be common themes in human tumours and are linked to poor prognosis and metastasis. Reduced expression of ASPP1 and ASPP2, or increased expression of iASPP, appear to be mechanisms involved in preventing wild-type p53 from working effectively. Hence, the ASPP family of proteins may be novel prognostic markers for human cancers. Identification of molecules that target the altered expression of the ASPP family members, whereby ASPP1 and ASPP2 expression could be enhanced or iASPP expression reduced, will allow us to develop new therapeutic strategies to treat cancer. For instance, a nine amino-acid peptide derived from ASPP2 (CBP3) was found to bind to the p53 core domain and stabilise it *in vitro* (Friedler *et al*, 2002). CBP3 was capable of restoring the apoptotic function of mutant p53, thereby killing mutant p53-expressing cells. From a chemical library screen, a small molecule, RITA (reactivation of p53 and induction of tumour cell apoptosis), was identified, which bound to p53 and induced its accumulation in tumour cells (Issaeva *et al*, 2004). RITA was shown to disrupt the interaction between p53 and MDM2, and was able to reactivate p53 in tumours that have aberrant MDM2 expression. The compound was also able to inhibit the interaction between iASPP and p53 and hence induce apoptosis.

The role of ASPP1, ASPP2 and iASPP in exerting positive and negative effects on the activity of p53 needs to be further explored in a tissue- and cell-type context. The altered expression of p53 activators such as ASPP1 and ASPP2 and inhibitors of p53 such as iASPP during tumour development in different tissue and cell types may explain the variation in p53 mutation rates in different tumour types. Furthermore, by taking into account both ASPP expression pattern and p53 codon 72 polymorphism, specific chemotherapeutic regimes could be tailored to different tumour types and individual patients.

## Figures and Tables

**Figure 1:**
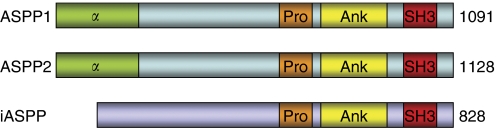
The human ASPP family structure. The amino-acid length of each ASPP member is indicated on the right, and individual structural elements are highlighted. Abbreviations: *α*, *α*-helical domain; Pro, proline-rich region; Ank, ankyrin repeats; SH3, SH3 domain.
